# GM-CSF does not rescue poor-quality embryos: secondary analysis of a randomized controlled trial

**DOI:** 10.1007/s00404-020-05532-3

**Published:** 2020-04-09

**Authors:** Kenny A. Rodriguez-Wallberg, Bibi Munding, Søren Ziebe, Sarah A. Robertson

**Affiliations:** 1 Division of Gynecology and Reproduction, Department of Reproductive Medicine, Karolinska University Hospital, Stockholm, Sweden; 2 Department of Oncology-Pathology, Karolinska Institute, Stockholm, Sweden; 3 CooperSurgical, ORIGIO a/S, Måløv, Denmark; 4 The Fertility Clinic, Rigshospitalet, University Hospital of Copenhagen, Copenhagen, Denmark; 5 Robinson Research Institute, Adelaide Medical School, University of Adelaide, Adelaide, SA Australia

**Keywords:** In vitro fertilization, Granulocyte–macrophage colony-stimulating factor (GM-CSF), Embryo culture, Implantation, Pregnancy rate, Live birth rate, Cleavage-stage embryos

## Abstract

**Purpose:**

To evaluate implantation potential of cleavage-stage embryos cultured in medium containing 2 ng/ml granulocyte–macrophage colony-stimulating factor (GM-CSF) versus control medium, according to embryo morphological quality and then transferred on day 3.

**Methods:**

Explorative secondary data analysis of a multicenter, randomized, placebo-controlled, double-blinded prospective study of 1149 couples with embryo transfer after IVF/ICSI. This analysis includes a subgroup of 422 subjects with either single-embryo transfer (SET, *N* = 286) or double-embryo transfer of two embryos with equivalent morphological quality (DET, *N* = 136). Implantation rate and live birth rate were assessed according to category of morphological embryo quality on day 3.

**Results:**

Culture with GM-CSF did not increase the implantation rate for embryos classified as poor quality. A trend towards greater benefit of GM-CSF on implantation and survival until live birth for top-quality embryos (TQEs) compared with poor-quality embryos was observed, although not statistically significant. For TQEs, the percentage of transferred embryos resulting in a live born baby was: 40.9 ± 5.3% (GM-CSF) versus 30.5 ± 4.6% (control) (*P* = 0.24; odds ratio [OR] 1.43, 95% confidence interval [CI] 0.79–2.59), and for embryos with less than 6 cells at day 3 this same rate was: 7.4 ± 3.3% (GM-CSF) versus 12.0 ± 4.0% (control) (*P* = 0.26; OR 0.53, 95% CI 0.17–1.61).

**Conclusion:**

This exploratory analysis is consistent with GM-CSF protecting morphologically normal embryos from culture-induced stress and does not support an effect of GM-CSF in rescuing poor-quality embryos.

ClinicalTrials.gov identifier: NCT00565747.

## Introduction

Although current in vitro conditions for culture of human embryos attempt to mimic the in vivo environment, it is generally recognized that the achieved conditions are suboptimal, and ideal culture media for in vitro development of human embryos are yet to be developed [1, 2].

In the female reproductive tract, several of the cytokines and growth factors secreted by the lining epithelium of the human fallopian tube and uterus have demonstrated effects in promoting normal embryo development and supporting embryo implantation [3]. Granulocyte–macrophage colony-stimulating factor (GM-CSF, also known as CSF2) is a lympho-hemopoietic cytokine with well-defined effects on proliferation and differentiation of different cell types. The expression of GM-CSF and its receptors in the human reproductive tract was first reported in the early nineties [4]. Later, it was demonstrated that GM-CSF increased significantly during a normally developing pregnancy, as estimated in plasma concentration levels, while that did not occur in women suffering from recurrent miscarriages [5]. These observations correspond well with data from animal studies, where GM-CSF deficient mice were found to have inherent fertility impairment [6], and abortion-prone mice could maintain gestation with improved neonatal outcome after treatment with exogenous GM-CSF [7]. Addition of GM-CSF to mouse embryo culture media has been shown to increase the proportion of transferred embryos generating viable progeny, and to partly alleviate the long-term adverse effects on fetal and postnatal growth after in vitro embryo culture [8].

The protective effects of GM-CSF identified for murine embryo culture include enhanced embryo viability by reduced cellular stress response and apoptosis [3, 9], and improved blastocyst development [10–14]. Similar beneficial effects of GM-CSF on development to blastocyst stage in embryo culture media have been observed in pigs, cattle and other livestock species [15–21] as well as in human embryos [22, 23]. In human studies, addition of GM-CSF to frozen/thawed human cleavage-stage embryos (2–4 cells) significantly improved rates of blastocyst development, from 30% in control to 76% in the GM-CSF group [22], and blastocysts cultured in the presence of GM-CSF were found to have 50% fewer apoptotic nuclei and 30% more viable inner cell mass cells [23]. However, data have been scarce with regard to the effect of GM-CSF on fresh cleavage-stage embryo development [24, 25].

A prospective randomized, placebo-controlled, double-blinded multicenter trial reported by Ziebe et al. [26], for which we here present a secondary explorative analysis, found that addition of GM-CSF to embryo culture medium elicited a significant increase in survival of transferred embryos to week 12 and live birth. This obvservation suggests that supplementation of culture medium with GM-CSF may increase developmental competence of pre-implantation cleavage-stage embryos [26]. Moreover, an exploratory analysis showed that GM-CSF increased embryo implantation rates in women who had experienced previous miscarriage, especially in women with a history of more than one miscarriage [26]. Similar results of improved ongoing pregnancy rates after three days of embryo culture in GM-CSF medium were obtained in a small study including 43 women with a history of previous miscarriage and/or implantation failure [27].

Regarding the possible mechanisms involved, a report by Karagenc et al. in mouse embryos [12] suggested that the effect of GM-CSF is more evident in embryos exposed to higher culture-induced stress achieved by virtue of reduced albumin content in the culture medium. This postulate is supported by further explorative analysis reported by Ziebe et al. [26] where subgroups were distinguished by different concentrations of human serum albumin (HSA) (2 mg/ml and 5 mg/ml, respectively) in both the GM-CSF and control medium, used in an initial and subsequent later phase of the trial, respectively. The largest effect of GM-CSF was observed when the medium contained 2 mg/ml HSA. However, independent of the HSA concentration in the culture media, Ziebe et al. found no significant differences in the morphology of embryo development in the GM-CSF versus control media [26].

In this explorative secondary analysis of data generated during the multicenter prospective randomized trial reported by Ziebe et al. [26], we aimed to investigate whether culture of human cleavage-stage embryos until day 3 in the presence of GM-CSF exerts beneficial effects on embryo implantation rate through differentially elevating the implantation competence of poor-quality embryos, or alternatively whether effects are exerted on embryos regardless of quality as judged by morphological characteristics.

## Materials and methods

### Study design and participants

This is an explorative secondary analysis of a subset of data from a previously reported prospective randomized, placebo-controlled, double-blinded, multicenter trial (ClinicalTrials.gov identifier: NCT00565747) [26]. Briefly, between April 2009 and August 2010, 528 women were enrolled at 13 Scandinavian fertility clinics, 11 Danish and 2 Swedish. The women were randomized to have their embryos cultured in the same culture medium without cytokine (control) or formulated with 2 ng/ml GM-CSF (test). Both media were produced by ORIGIO a/s and with optimized concentration of human serum albumin (5 mg/ml). This report focuses on a subgroup of 422 women characterized by having a single-embryo transfer (SET) (*N* = 286) or a double-embryo transfer (DET) (*N* = 136) of two embryos of comparable morphological quality. Women transferred with two embryos of different morphological quality (*N* = 106) were excluded from this investigation.

### Study procedures

Details on study design and randomization procedure have been described previously [26]. Briefly, patients were eligible for inclusion if they were aged 25–39 years, had a regular menstrual cycle of 21–35 days, were treated with a standard GnRH agonist or antagonist protocol, and had three or more follicles with a diameter of > 17 mm on the day of hCG administration. Exclusion criteria were previous participation in the study, use of assisted hatching, use of non-ejaculated sperm, medical conditions or genetic disorders prohibiting IVF/ICSI or interfering with interpretation of results, use of investigational drugs within 30 days before oocyte retrieval, severe chronic disease of relevance for reproduction/implantation, and oocyte donation.

### Sample size and data analysis

Embryos were categorized by morphological score, with ‘Abnormally Developed’ embryos (ADE) having < 6 cells at 68 h post-insemination, ‘Normally Developed’ embryos (NDE) having ≥ 6 cells and ≤ 20% fragmentation at 68 h post-insemination, without fulfilling the criteria for Top Quality Embryo (TQE); and TQE having 4–5 cells at 44 h post-insemination, ≥ 7 cells at 68 h post-insemination, ≤ 20% fragmentation, equally sized blastomeres with < 25% difference, and no signs of multinucleation.

The effect of GM-CSF on embryo implantation was investigated by exploratory statistical analysis to evaluate trends with regard to embryo implantation rate and survival until live birth when comparing embryos of comparable morphology. Data analysis were performed for a combined group of women with single-embryo transfer or double-embryo transfer with embryos of equivalent morphological quality (*N* = 422 subjects). Odds ratios (ORs) for the effect parameters (ongoing implantation rate gestational week 7 and week 12, and live babies born per embryo transferred) were analyzed with the use of a logistic regression model (SAS software version 9.2) with treatment group (GM-CSF/control) as main factor, and covariates including age, smoking status and number of oocytes collected. Age, BMI and number of oocytes collected were compared between GM-CSF and control group using the *t* test. All categorical comparisons were performed using Fisher’s exact test.

## Results

Patient characteristics and clinical outcome by group assigned to transfer of embryo(s) cultured in GM-CSF or control medium are presented in Tables 1 and 2. There were no statistically significant differences between test and control groups with regard to age or BMI of the women, cause of infertility, IVF or ICSI insemination procedures, or number of oocytes obtained.

**Table 1:** Clinical characteristics of the study population (single-embryo transfer or double-embryo transfer with embryos of equivalent morphological category; *N* = 422 subjects)

	GM-CSF	Control	*P* value
Subjects/transfer cycles (*N*)	206	216	–
Age of woman (years)	32.5 ± 3.8	32.0 ± 3.8	0.14
Age of woman < 35 year [*N* (%)]	133 (64.6)	155 (71.8)	0.12
Age of woman ≥ 35 year [*N* (%)]	73 (35.4)	61 (28.2)	
BMI (kg/m^2^)	24.3 ± 4.4	24.5 ± 4.4	0.65
Cause of infertility [*N* (%)]			
Male factor	96 (46.6)	96 (44.4)	0.70
Unexplained infertility	62 (30.1)	68 (31.5)	0.83
Tubal disease	32 (15.5)	34 (15.7)	1.00
PCO ovaries	6 (2.9)	7 (3.2)	1.00
Endometriosis	21 (10.2)	13 (6.0)	0.15
Uterine factor	6 (2.9)	2 (0.9)	0.17
Other^a^	7 (3.4)	12 (5.6)	0.35
Previous IVF/ICSI [*N* (%)]	98 (47.5)	98 (45.4)	0.70
Previous pregnancy [*N* (%)]	85 (41.3)	88 (40.7)	0.91
Previous births [*N* (%)]	41 (19.9)	45 (20.8)	0.90
Previous miscarriage [*N* (%)]	49 (23.8)	52 (24.1)	1.00
Fertilization procedure [*N* (%)]			
IVF	110 (53.4)	108 (50.0)	0.55
ICSI	77 (37.4)	80 (37.0)	
Both	19 (9.2)	27 (12.5)	
Oocytes retrieved	10.0 ± 4.8	10.5 ± 4.8	0.27

Plus–minus values are mean ± standard deviation (SD). Age, BMI and number of oocytes collected were compared between GM-CSF and Control using the *t* test. All categorical comparisons were performed using Fisher’s exact test

^a^Other: cases which cannot be categorized as one of the standard indications (e.g., single, lesbian, etc.)

**Table 2:** Clinical outcomes for the study population (single-embryo transfer or double-embryo transfer with embryos of equivalent morphological category; *N* = 422 subjects)

	GM-CSF	Control	*P* value
Subjects/transfer cycles (*N*)	206	216	–
Number of embryos transferred (*n*)	274	284	–
Mean no. of embryos transferred	1.33	1.31	–
Supernumerary embryos frozen [*n*_embryos_ (% of embryos obtained)	390 (18.8)	484 (20.3)	0.17
Ongoing implantation rate week 7 (% of transferred embryos)	26.0 ± 3.0	25.2 ± 2.8	0.18
Ongoing implantation rate week 12 (% of transferred embryos)	26.0 ± 3.0	23.4 ± 2.7	0.095
Live babies born (% of transferred embryos)	26.0 ± 3.0	23.4 ± 2.7	0.095
Follow-up on pregnancies			
Positive hCG [*N* (% of transfer cycles)]	74 (35.9)	89 (41.2)	0.55
Biochemical pregnancy^a^ [*N* (% of women with positive hCG)]	13 (6.3)	17 (7.9)	0.57
Early pregnancy loss (≤ week 12) [*N* (% of women with positive hCG)]	1 (0.5)	13 (6.0)	0.0016
Late pregnancy loss (> week 12) [*N* (% of women with positive hCG)]	1 (0.5)	4 (1.9)	0.37
Live birth [*N* (% of transfer cycles)]	58 (28.2)	54 (25.0)	0.54

Plus–minus values are rates ± standard error (s.e). All categorical comparisons were performed using Fisher’s exact test. Analysis of positive hCG, implantation rates and live birth were performed using a logistic regression model with treatment (GM-CSF/control) as main factor, and covariates including embryo quality transferred, woman’s age, smoking status and number of oocytes collected

^a^Biochemical pregnancy: positive hCG but no ultrasound verification of an intrauterine gestational sac

The clinical success rates after IVF/ICSI and embryo culture and transfer in GM-CSF and control medium, respectively, according to the morphological category of the embryo(s) transferred, are shown in Table 3. For the subgroups of embryos categorized as having the highest morphological quality (TQE), data show tendencies to improved embryo survival until live birth after culture and transfer in GM-CSF medium, with the percentage of transferred embryos resulting in a live born baby being 40.9 ± 5.3% (GM-CSF) versus 30.5 ± 4.6% (control) (*P* = 0.24; odds ratio [OR] 1.43, 95% confidence interval [CI] 0.79–2.59).

**Table 3:** Clinical success rates after single- or double-embryo transfer (SET + DET); according to the morphological categorization of the embryos transferred

	Abnormal embryo		*P* value (OR [95% CI])	Normal embryo		*P* value (OR [95% CI])	TQE		*P* value (OR [95% CI])
	GM-CSF	Control		GM-CSF	Control		GM-CSF	Control	
# Subjects (*N*)	54	54	–	70	67	–	82	95	–
# Embryos (*n*)	85	85	–	92	84	–	97	115	–
Embryo data									
Ongoing implantation rate week 7	7.4 ± 3.3	12.0 ± 4.0	0.26 (0.53 [0.17–1.61])	22.9 ± 4.8	25.4 ± 5.1	0.88 (1.06 [0.50–2.25])	40.9 ± 5.3	32.6 ± 4.7	0.35 (1.33 [0.73–2.40])
Ongoing implantation rate week 12	7.4 ± 3.3	12.0 ± 4.0	0.26 (0.53 [0.17–1.61])	22.9 ± 4.8	22.4 ± 4.9	0.63 (1.21 [0.56–2.61])	40.9 ± 5.3	30.5 ± 4.6	0.24 (1.43 [0.79–2.59])
Live babies per embryo transferred	7.4 ± 3.3	12.0 ± 4.0	0.26 (0.53 [0.17–1.61])	22.9 ± 4.8	22.4 ± 4.9	0.63 (1.21 [0.56–2.61])	40.9 ± 5.3	30.5 ± 4.6	0.24 (1.43 [0.79–2.59])
Patient data									
Ongoing clinical pregnancy rate week 7	9.3 ± 4.0	16.7 ± 5.1	0.26 (0.49 [0.14–1.67])	25.7 ± 5.3	28.4 ± 5.5	0.88 (1.06 [0.48–2.34])	43.9 ± 5.5	35.8 ± 4.9	0.28 (1.41 [0.76–2.63])
Ongoing clinical pregnancy rate week 12	9.3 ± 4.0	16.7 ± 5.1	0.26 (0.49 [0.14–1.67])	25.7 ± 5.3	25.4 ± 5.4	0.61 (1.24 [0.55–2.76])	43.9 ± 5.5	33.7 ± 4.9	0.18 (1.53 [0.82–2.86])
Live birth per subject	9.3 ± 4.0	14.8 ± 4.9	0.41 (0.59 [0.17–2.05])	25.7 ± 5.3	23.9 ± 5.2	0.50 (1.32 [0.59–2.99])	42.7 ± 5.5	31.6 ± 4.8	0.13 (1.64 [0.87–3.09])

Plus–minus values are rates ± standard error (s.e)

Embryo data are percentages calculated per number of embryos transferred

Patient data are percentages calculated per number of women with embryo transfer

*P* probability in a 95% confidence interval, *OR* odds ratio

However, no beneficial effect of culture with GM-CSF was observed in the remaining subgroups of abnormally (ADE) and normally developed embryos (NDE). In contrast, a tendency to decreased implantation potential was observed for the group of embryos with poor morphological quality (ADE) when cultured in GM-CSF medium, with the percentage of transferred embryos resulting in a live born baby being 7.4 ± 3.3% (GM-CSF) versus 12.0 ± 4.0% (control) (*P* = 0.26; OR 0.53, 95% CI 0.17–1.61). Moreover, there was no effect of GM-CSF on the group of NDE’s (embryos of transferable quality but not fulfilling the criteria for TQE): with the percentage of transferred embryos resulting in a live born baby being 22.9 ± 4.8% (GM-CSF) versus 22.4 ± 4.9% (control) (*P* = 0.63; OR 1.21, 95% CI 0.56–2.61).

No differences between GM-CSF and control groups were observed with regard to stillbirth, perinatal death, abnormalities or birth weight of babies born (data not shown).

## Discussion

Here, we report an exploratory secondary analysis investigating a subgroup of patients participating in a multicenter prospective randomized trial reported by Ziebe et al. [26]. The subgroup is characterized by being enrolled in the late phase of the trial where all embryos were cultured in either test (GM-CSF) or control medium (without GM-CSF) with optimized concentration of human serum albumin (5 mg/ml). Secondly, the patient population was limited to those with single-embryo transfer or double-embryo transfer (DET) of two embryos of comparable morphological quality.

Although statistical significance was not reached due to insufficient power for this exploratory subgroup analysis, it was a consistent observation for all success parameters both on the embryo level and patient level that the presence of GM-CSF reduced favorable outcomes in the poor embryo quality group; while in the highest embryo quality group, GM-CSF exhibited favorable effects. In the intermediate group, no effect of GM-CSF was observed on the success parameters. These data suggest that the improved implantation rates and progression to live birth found by Ziebe et al. [26] is principally the result of effects of GM-CSF on morphologically normal as opposed to morphological abnormal embryos.

Importantly, this analysis alleviates any concerns that pro-survival cytokines may cause embryos otherwise destined for demise, to survive. It has been suggested that GM-CSF might rescue developmentally compromised embryos, potentially resulting in implantation and ongoing development of embryos that would not have survived in the absence of cytokine [28]. Our exploratory analysis does not support the interpretation that GM-CSF acts to rescue inferior or damaged embryos that could be associated with impaired fetal development and/or poor neonatal outcomes.

The current data also imply that beneficial effects of GM-CSF cannot be attributed to rescuing human embryos from poor or highly stressful culture conditions [12], as all embryos were cultured in test (2 ng/ml GM-CSF) or control medium, both with optimized concentration of human serum albumin (5 mg/ml).

Our rationale for this analysis was to search for evidence of GM-CSF effects acquired by the cleavage-stage embryo during culture. Effects of GM-CSF that are evident at blastocyst stage, particularly reduced incidence of apoptosis and elevated cell number [22], might become evident if cleavage-stage embryos were cultured for a longer period, but were not evident in this study as embryos were transferred on day 3 of embryo culture. Our observations provide support for the argument that embryo exposure to pro-survival cytokines exert beneficial effects on implantation potential without necessarily being reflected in effects on cleavage-stage embryo morphology.

This result adds to previous evidence that exposure of human embryos to GM-CSF in culture does not increase the likelihood of stillbirth, perinatal death, congenital abnormalities or abnormal birth weight of singletons and twins when compared with control medium without GM-CSF [26]. The result is also consistent with previous data showing similar chromosomal constitution of human embryos cultured in vitro with or without the presence of 2 ng/ml GM-CSF [25].
